# HtrA Contributes to Biofilm Formation in *Mycobacterium smegmatis* by Downregulating the Cell Wall Amidase Ami3

**DOI:** 10.3390/microorganisms13122688

**Published:** 2025-11-25

**Authors:** Jiachen Zheng, Yueqi Li, Yizhang Wei, Kang Li, Jie Lu, Xiaolin Liu, Weihui Li

**Affiliations:** College of Life Science and Technology, Guangxi University, Guangxi Research Center for Microbial and Enzyme Engineering Technology, State Key Laboratory for Conservation and Utilization of Subtropical Agro-Bioresources, Nanning 530004, China

**Keywords:** trypsin, biofilm, cell wall amidase, mycobacteria

## Abstract

*Mycobacterium tuberculosis*, the causative agent of tuberculosis, utilizes biofilm formation as a key mechanism to withstand host-derived stresses. To identify novel factors involved in this process, we performed a CRISPRi screen in the model organism *Mycobacterium smegmatis*. This screen identified trypsin HtrA as a critical factor for growth and biofilm formation. Deletion of *htrA* led to a profound upregulation of the cell wall amidase Ami3. We demonstrated that Ami3 is a crucial negative regulator of biofilm formation, as overexpression of *ami3* recapitulated the biofilm and growth defects of the Δ*htrA* strain. Furthermore, we found that the essential role of periplasmic protease HtrA for normal growth could be suppressed by novel mutations in *pmt*, a gene encoding a phosphomyoinositol mannosyltransferase, at residues F53 and N55, distinct from the previously reported D68 site. Our findings establish a novel regulatory pathway in which HtrA modulates mycobacterial biofilm formation by controlling the levels of Ami3 and reveal new genetic interactions within this network.

## 1. Introduction

Tuberculosis (TB), caused by the pathogenic bacterium *Mycobacterium tuberculosis*, remains a major global health challenge. A significant factor contributing to the persistence of this disease is the ability of *M. tuberculosis* to form biofilms [1,2,3]. The biofilm is an aggregate of microorganisms in which cells are adhered to each other or to a surface and are enclosed within a self-produced matrix of extracellular polymeric substances (EPS). On solid media, this process can be preliminarily assessed by colony appearance. A rough colony morphology signifies proficient biofilm development, while a smooth colony originates from a fundamental cell envelope defect that impedes biofilm formation altogether [4,5]. These structured communities of surface-adhered cells are encased in an extracellular matrix and exhibit coordinated behavior [6], resulting in enhanced tolerance against environmental stresses and antibiotics [7,8,9]. The unique, lipid-rich architecture of mycobacterial biofilms is a key contributor to the resilience of pathogenic strains, facilitating treatment failure and increasing the risk of relapse [10]. The formation of biofilms requires specific molecular components at different stages. For instance, in *M. smegmatis*, glycopeptidolipids are considered crucial for initial attachment [11], and mycolic acids are important for structural development, a process facilitated by the Hsp60 chaperone GroEL1 [12]. However, it remains unknown whether critical regulatory pathways, particularly those involving proteolytic control of cell wall hydrolases, govern this process.

To endure the hostile environment within the host, *M. tuberculosis* employs sophisticated stress response mechanisms, including proteases that play an indispensable role in maintaining cellular homeostasis, supporting growth, and promoting virulence. A key member of this proteolytic network is the high-temperature requirement A (HtrA) protein, a highly conserved protease. First identified in *Escherichia coli* for its essential role at high temperatures [13], HtrA family proteins are involved in diverse cellular processes [14]. Structurally, HtrA proteases feature a trypsin-like catalytic domain and a regulatory PDZ domain [15]. Their primary function is protein quality control, degrading misfolded proteins in the periplasm [16,17,18]. Notably, some HtrA homologs, such as DegP in *E. coli*, also exhibit chaperone activity, stabilizing specific proteins and protecting cells from proteotoxic stress [19,20]. Beyond its housekeeping roles, HtrA is a significant virulence factor in many pathogens [21,22,23]. As a secreted protein, it can contribute to pathogenesis by cleaving the extracellular domain of E-cadherin in mammalian cells, a mechanism conserved in *Helicobacter pylori* [24], enteropathogenic *E. coli*, *Salmonella typhimurium*, *Campylobacter jejuni*, and *Pseudomonas aeruginosa* [25]. E-cadherin represents a mammalian cell surface protein that has essential functions in cell adhesion and tumor suppression. In gastric cancer, proteolytic release of the E-cadherin ectodomain is an important prognostic marker [26]. In mammalian cells, the matrix metalloproteases MMP3 and MMP7 and a disintegrin and metallopeptidase 10 were identified to cleave E-cadherin extracellularly, generating a soluble fragment that impairs cell adhesions [27,28].

The influence of HtrA on bacterial social behavior is important, particularly in biofilm formation. In *Streptococcus mutans* and *Vibrio cholerae*, HtrA is required for robust biofilm development, as its deletion leads to significant defects [24,29]. In contrast, in other species, the secreted form of HtrA has been shown to suppress biofilm development [30]. Given the critical role of biofilms in mycobacterial persistence, the function of HtrA in this process warrants investigation. However, the specific mechanism through which HtrA regulates biofilm formation in mycobacteria remains to be elucidated.

The *M. tuberculosis* genome encodes three HtrA paralogs: HtrA (Rv1223), PepD (Rv0983), and PepA (Rv0125). Among these, only HtrA is indispensable for bacterial survival [31], underscoring its critical and non-redundant function. A pivotal clue to HtrA’s essential role comes from studies in the model organism *M. smegmatis*, which revealed that HtrA regulates the cell wall amidase Ami3 [32]. HtrA as a periplasmic serine protease is essential in mycobacteria, which forms a periplasmic complex with another essential lipoprotein, LppZ, thereby exerting its function to control levels of the lethal hyperactivity of Ami3 [32]. In *Escherichia coli,* a member of the highly conserved HtrA family of proteases, DegP, the interaction between DegP and LppZ results in the stabilization of specific conformations that can be either inactive or active [33]. These examples demonstrate how lipoproteins act as allosteric regulators of proteases in diverse biological systems [34]. These results demonstrate that LppZ is essential for HtrA function. It has been proposed that in the absence of HtrA, Ami3 accumulates to toxic levels when stabilized by mannosylation mediated by the phosphatidylinositol mannosyltransferase (Pmt) [32], suggesting that HtrA-mediated degradation of Ami3 is vital for cell viability. Protein-O-mannosyltransferases was first discussed in yeast [35]. It has become apparent that Pmt is ubiquitous across the evolutionary spectrum, from prokaryotes to eukaryotes. Such as in bacteria, O-glycosylated proteins were found in flagellae (flagellin), pili (pilin) and other secretory proteins [35]. The formation of hydrophobic structures by Pmt on the *C. albicans* cell surface plays an important role in biofilm biogenesis [35]. Previous studies have shown that Pmt enhances the stability of Ami3 via mannosylation [32]. We therefore speculate that the mutation of Pmt may compromise its function, leading to reduced stability of Ami3, which in turn facilitates the knockout of *htrA*. Mycobacterial genomes encode four putative peptidoglycan-degrading amidases: Ami1 (Rv3717), Ami2 (Rv3915), Ami3 (Rv3811), and Ami4 (Rv3594), but their specific roles, particularly in biofilm formation, are not well defined [36,37,38].

In this study, we leveraged a CRISPRi (CRISPR interference) screen in *M. smegmatis* to identify HtrA as a critical factor for growth and biofilm formation. Subsequent proteomics analysis revealed significant upregulation of Ami3 in *htrA* knockout strains. Our findings establish that HtrA regulates biofilm formation in *M. smegmatis* through the cell wall amidase Ami3. Furthermore, while previous studies identified a specific mutation in Pmt (D68A) as a prerequisite for the successful knockout of the *htrA* [32,37,39], we discovered novel compensatory mutations in *pmt*. Overall, our findings establish a direct link between HtrA and Ami3 in the regulation of mycobacterial biofilm formation and unveil new genetic interactions within this essential pathway.

## 2. Results

### 2.1. HtrA Is Essential for Growth and Biofilm Formation in M. smegmatis

**Figure 1:**
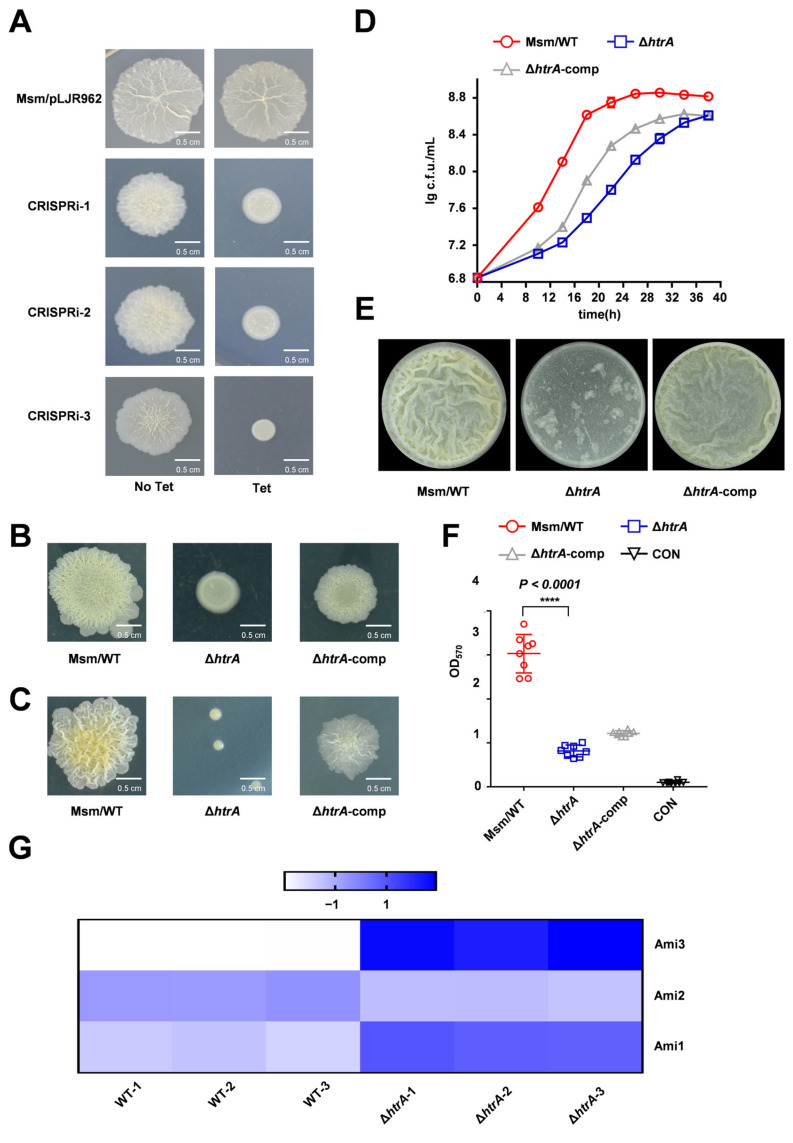
**HtrA is required for normal growth, colony morphology, and biofilm formation in *M. smegmatis*.** (**A**) Colony morphology of wild-type (WT) and three independent CRISPRi strains targeting *htrA* (CRISPRi-1/2/3) following tetracycline induction, as shown by spot assay. (**B**) Colony morphology of WT, Δ*htrA*, and the complemented strain (Δ*htrA*-comp) on spot plates. (**C**) Colony morphology of WT, Δ*htrA*, and the complemented strain (Δ*htrA*-comp) were serially diluted cultures plated for single-colony morphology. (**D**) Growth curves of WT, Δ*htrA*, and the complemented strain under standard culture conditions. Data represent mean ± SD of at least three biological replicates. (**E**) Representative images of air-liquid interface biofilms formed by WT, Δ*htrA*, and the complemented strain. (**F**) ‘CON’ is defined as ‘medium-only control.’ Quantification of biofilm biomass by crystal violet staining. Data represent mean ± SD of at least eight biological replicates. ****, *p* < 0.0001 compared to WT. (**G**) Heatmap of normalized protein levels for Ami1, Ami2, and Ami3 from quantitative proteomic analysis of WT and Δ*htrA* strains. (The color scale from −1 to 1 represents expression levels relative to the mean (blue: high, white: low)).

To identify novel genes involved in biofilm formation, we performed a whole-genome CRISPRi screen in *M. smegmatis*. In this model organism, colony morphology is a direct and causal indicator of biofilm capability [11,40]. We selected knockdown strains with altered colony morphology for sequencing, which identified the serine protease gene *htrA* as a candidate. Upon tetracycline induction, three independent CRISPRi strains targeting *htrA* all exhibited significant alterations in colony surface morphology compared to the wild-type (WT) strain (Figure 1A). The WT strain formed a typical wrinkled colony that was rougher and larger than the smooth and small colonies of the *htrA* (knock-down) strains (Figure 1A). To confirm this phenotype, we constructed an *htrA* knockout mutant using homologous recombination. The Δ*htrA* strain exhibited distinct colony morphology on both spot and serially diluted cultures plated for single-colony morphology, a phenotype that was partially rescued in the genetic complementation strain (Figure 1B,C). Growth curve analysis further revealed that the *htrA* knockout strain exhibited a slower growth rate compared to the wild-type, which was restored upon complementation (Figure 1D). Finally, using gas–liquid interface biofilm assays and crystal violet quantification, we found that the deletion of *htrA* severely impaired biofilm formation in *M. smegmatis* (Figure 1E,F). The crystal violet staining visually confirms that the phenotypic restoration is achieved through *htrA* complementation (Figure S1). To rule out the possibility that the biofilm defect in the Δ*htrA* strain was solely a consequence of its growth impairment, we extended the incubation period for the gas–liquid interface biofilm assay to 72 h. The Δ*htrA* strain still failed to form a robust biofilm under these conditions, while the wild-type biofilm was fully developed. This indicates that the biofilm impairment is a direct physiological consequence of the *htrA* deletion (Figure S2).

Collectively, these results establish that HtrA is critical for normal colony morphology, growth, and biofilm development in *M. smegmatis*. Together with the observed colony morphology defects, our results demonstrate that HtrA is critical for normal colony morphology, growth, and biofilm formation in *M. smegmatis*.

### 2.2. HtrA Modulates Mycobacterial Biofilm Formation Via Ami3

**Figure 2:**
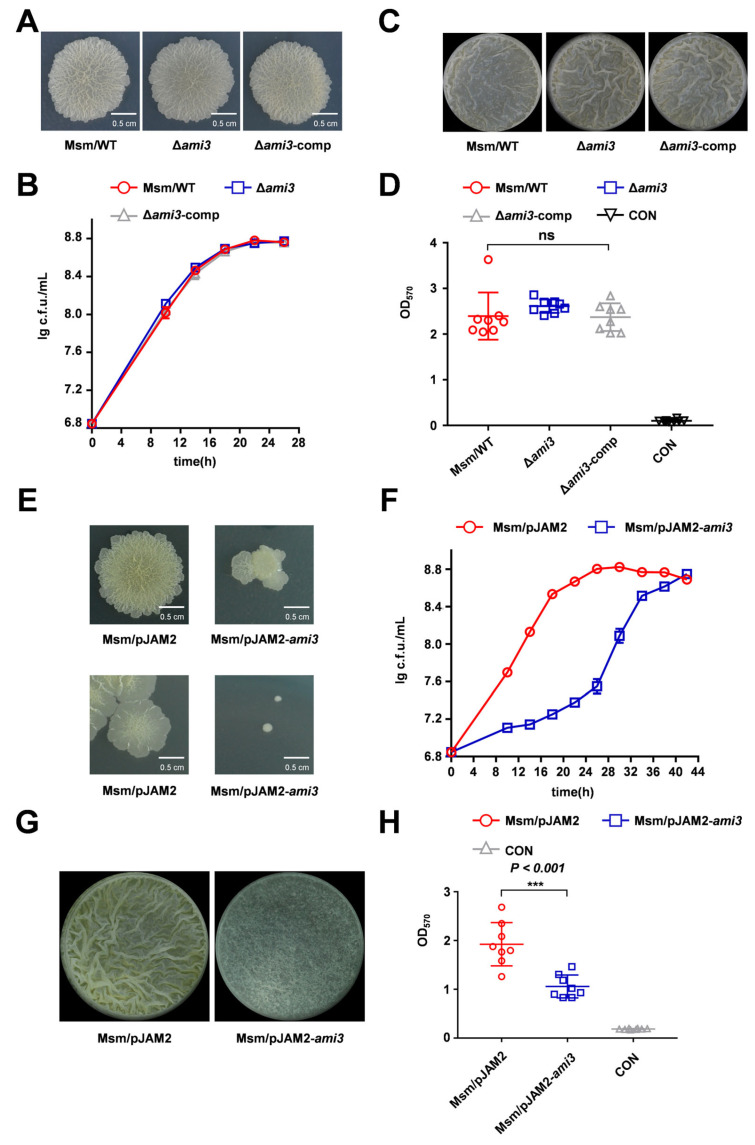
**Overexpression of Ami3 recapitulates the Δ*htrA* phenotype.** (**A**) Colony morphology of wild-type (WT), Δ*ami3*, and Δ*ami3*-comp strains assessed by spot assay. (**B**) Growth curves of WT, Δ*ami3*, and Δ*ami3*-comp strains under standard culture conditions. Data represent mean ± SD of at least three biological replicates. (**C**) Representative images of air-liquid interface biofilms formed by WT, Δ*ami3*, and Δ*ami3*-comp strains. (**D**) Quantification of biofilm biomass for WT, Δ*ami3*, and Δ*ami3*-comp by crystal violet staining. Data represent mean ± SD of at least eight biological replicates. (**E**) Comparison of differences in colony morphology between Msm/pJAM2-*ami3* and WT on spot plates (top panel), and Msm/pJAM2-*ami3* and WT on dilution plate (bottom panel). (**F**) Growth curves of WT and the *ami3* overexpression strain under normal conditions. (**G**) Representative images of air-liquid interface biofilms formed by Msm/pJAM2-*ami3* and WT. (**H**) Quantification of biofilm biomass for Msm/pJAM2-*ami*3 and WT strain by crystal violet staining. Data represent mean ± SD of at least eight biological replicates. Statistically significant differences are represented as *** (*p* < 0.001, ns, not significant).

To identify the downstream effectors of HtrA regulating biofilm formation in *M. smegmatis*, we performed quantitative proteomic analysis on the Δ*htrA* strain (Tables S1 and S2). This approach revealed a striking upregulation of the cell wall amidase Ami3, which increased by 121.957-fold in Δ*htrA* strain, while Ami2 levels were slightly decreased 0.625-fold (Figure 1G). To determine the functional consequence of Ami3 loss, we constructed an *ami3* knockout strain (Δ*ami3*) in *M. smegmatis*. Phenotypic characterization revealed that the Δ*ami3* strain exhibited colony morphology, biofilm formation profiles and its growth profile same as WT (Figure 2A–C). Given that the expression of *ami3* was significantly increased in the Δ*htrA* strain, we hypothesized that Ami3 overexpression is the primary driver of the observed phenotypes in the Δ*htrA* strain. To test this hypothesis, we constructed an *ami3* overexpression strain in WT background. Remarkably, this strain recapitulated the hallmark features of the Δ*htrA* mutant, including altered colony morphology, impaired growth, and defective biofilm formation (Figure 2E–H). To further test the hypothesis that Ami3 hyperactivity is responsible for the Δ*htrA* phenotypes, we performed a genetic knockdown of *ami3* in Δ*htrA* strain. The knockdown of *ami3* partially rescued the growth and biofilm formation defects in the *htrA* knockout mutant (Figures S3 and S4).

These results demonstrate that the biofilm and growth deficiencies caused by *htrA* deletion are associated with the accumulation of Ami3.

### 2.3. Ami3 Is Remarkably Conserved Across Mycobacteria

**Figure 3:**
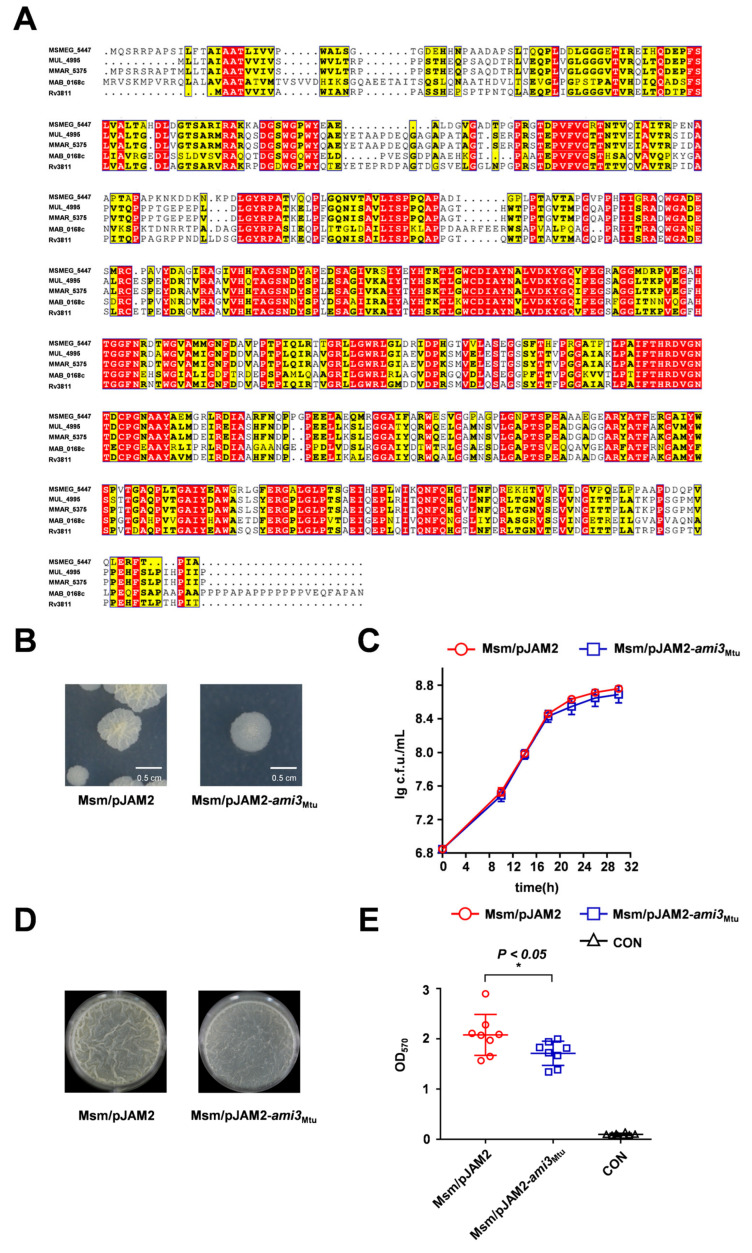
**Heterologous expression of *M. tuberculosis* Ami3 inhibits biofilm formation in *M. smegmatis*.** (**A**) Sequence alignment of Ami3 from *M. smegmatis* (Msmeg_5447), *M. tuberculosis* (Rv3811), *M. marinum* (MMAR_5375)*, M. ulcerans* (MUL_4995) and *M. abscessus* (MAB_0168c). Red indicates strictly conserved residues across all five mycobacterial species, yellow indicates semi-conserved substitutions (within the same amino acid class), and white indicates non-conserved residues. (**B**) Colony morphology of Msm/pJAM2-*ami3*_Mtu_ and WT, shown using a streaking assay. (**C**) Growth curves of Msm/pJAM2-*ami3*_Mtu_ and WT strains under standard culture conditions. Data represent mean ± SD of at least three biological replicates. (**D**) Representative images of air-liquid interface biofilms formed by Msm/pJAM2-*ami3*_Mtu_ and WT strains. (**E**) Quantification of biofilm biomass of Msm/pJAM2-*ami3*_Mtu_ and WT by crystal violet staining. Data represent mean ± SD of at least three biological replicates in growth curves and at least eight biological replicates in crystal violet staining quantitative assay. Statistically significant differences are represented as * (*p* < 0.05).

We next investigated whether the role of Ami3 is conserved in the major pathogen *M. tuberculosis*. Sequence alignment revealed 61.6% amino acid similarity between Ami3 from *M. smegmatis* (Ami3_Msm_) and *M. tuberculosis* (Ami3_Mtu_) (Figure 3A). To test functional conservation, we overexpressed *ami3*_Mtu_ in *M. smegmatis* WT strain. The resulting strain exhibited a smoother colony morphology on dilution plates, phenocopying the effect of *ami3*_Msm_ overexpression strain (Figure 3B). Interestingly, despite this morphological similarity, overexpression of *ami3*_Mtu_ did not recapitulate the growth defect associated with *ami3*_Msm_ overexpression (Figure 3C). Crucially, the *ami3*_Mtu_ overexpression strain showed a significant impairment in biofilm formation, as measured by both gas–liquid interface assays and crystal violet staining (Figure 3D,E).

These findings demonstrate that the core function of Ami3 as a negative regulator of biofilm formation is conserved in *M. tuberculosis*, while its pronounced effect on growth appears to be species-specific.

### 2.4. Ami3, but Not Ami2, Is a Key Regulator of Biofilm Formation in M. smegmatis

**Figure 4:**
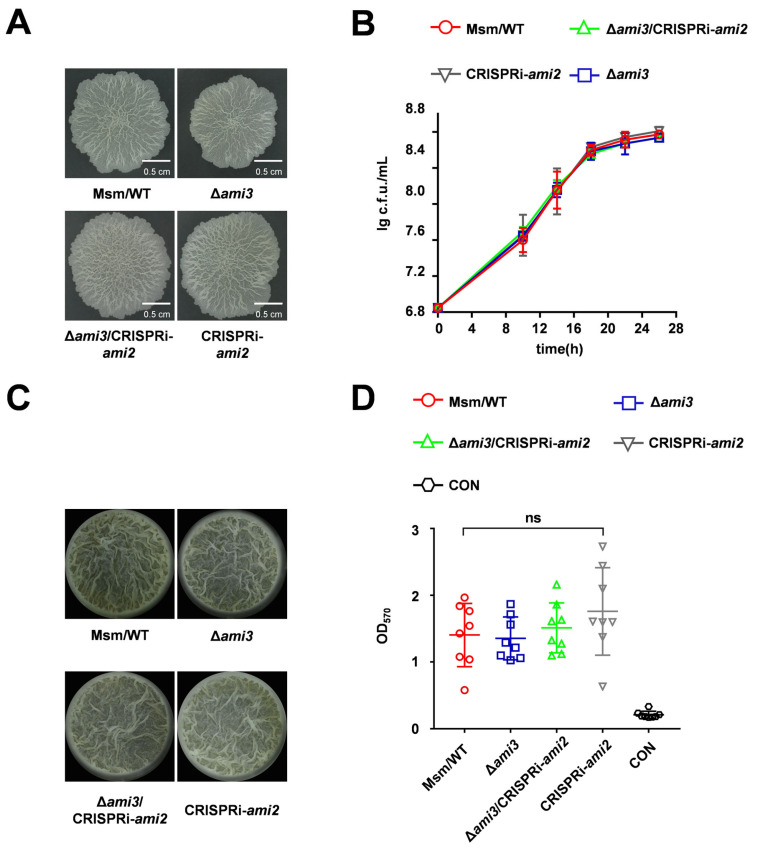
**Ami2 does not contribute to the HtrA-regulated biofilm phenotype.** (**A**) Colony morphology of WT, Δ*ami*3, Δ*ami*3/CRISPRi-*ami*2, and CRISPRi-*ami*2 strains, assessed by spotting plates assay. (**B**) Growth curves of the indicated strains under normal conditions. (**C**) Representative images of air-liquid interface biofilms formed by the indicated strains. (**D**) Quantification of biofilm biomass of WT, Δ*ami*3, Δ*ami*3/CRISPRi-*ami*2, and CRISPRi-*ami*2 by crystal violet staining. Data represent mean ± SD of at least three biological replicates in growth curves and at least eight biological replicates in crystal violet staining quantitative assay. Statistically significant differences are represented as (ns, not significant).

Proteomic analysis indicated that deletion of *htrA* led to alterations in both Ami3 and Ami2 levels. To investigate whether Ami2 downregulation contributes to the Δ*htrA* phenotype, we constructed an *ami2* knockdown mutant. This mutant showed no differences from the wild type in colony morphology or growth (Figure 4A,B). Furthermore, knocking down *ami2* in Δ*ami3* genetic background also failed to produce any changes in colony morphology or growth (Figure 4A,B). Quantitative biofilms confirmed that the severe biofilm deficiency in Δ*htrA* is independent of *ami2* downregulation (Figure 4C,D). Furthermore, these results collectively demonstrate that Ami3 is the central amidase regulating biofilm formation in this pathway.

### 2.5. Identification of Novel Suppressor Mutations in Pmt

**Figure 5:**
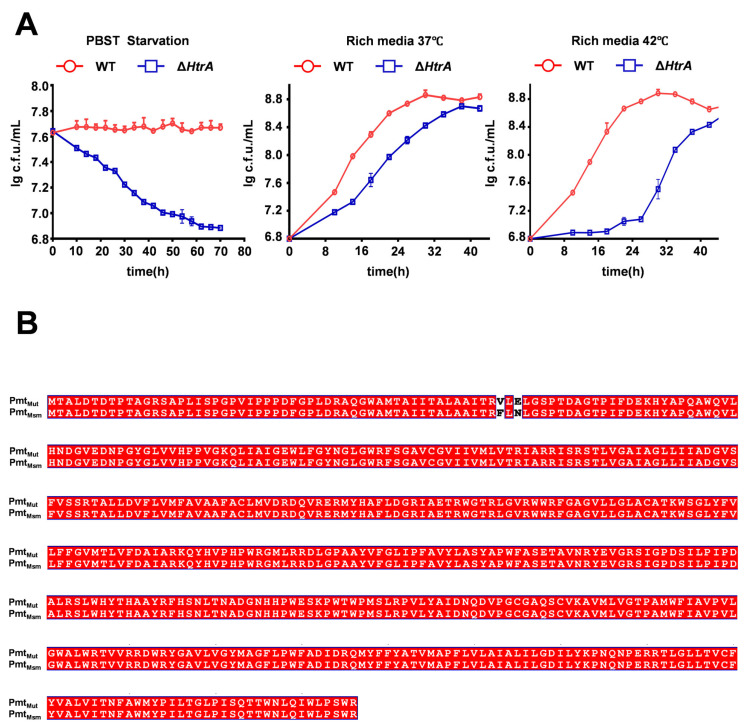
**The Δ*htrA* strain exhibits growth defects and harbors novel compensatory mutations in *pmt*.** (**A**) Growth curves of wild-type (WT) and Δ*htrA* strains under three conditions: rich media at 37 °C, high temperature (42 °C), carbon-nitrogen starvation (PBS-Tween 20). Δ*htrA* strain shows impaired growth under all conditions. Error bars represent SD of the mean. (**B**) Sequence alignment of Pmt from the wild-type (Pmt_Msm_) and the Δ*htrA* mutant (Pmt_mut_). The alignment confirms the acquisition of two novel mutations, F53V and N55E, in the Δ*htrA* background. Red denotes amino acid residues that are identical in the mutant and wild-type sequences. Conversely, white highlights the amino acid substitutions specific to the mutant.

The essential requirement for HtrA in mycobacteria can be bypassed by compensatory mutations in *pmt*, a gene encoding a phospho-myo-inositol mannosyltransferase, with a mutation at residue D68 being a known prerequisite for *htrA* deletion [32]. To confirm that the phenotypes of our Δ*htrA* strain were not masked by such suppressors, we first characterized its growth under various conditions. The Δ*htrA* strain exhibited impaired growth compared to the wild type under both starvation and nutrient-rich conditions at various temperatures, confirming the successful generation of a functional knockout (Figure 5A). We then sequenced *pmt* and *ami3* in this strain to identify any potential compensatory mutations. This analysis revealed novel mutations in Pmt at residue F53 and residue N55 sites distinct from the known position 68, while the *ami3* sequence remained wild-type (Figure 5B).

These results demonstrate that a novel mutation in Pmt, at a site distinct from the known residue 68, can also functionally compensate for the growth defect caused by *htrA* knockout.

## 3. Discussion

In this study, we identify a regulatory pathway in which the protease HtrA governs biofilm formation and growth in mycobacteria, possibly by controlling the stability of the cell wall amidase Ami3. Additionally, we investigated the relationship between other proteins in the Ami family and biofilms. Sequencing of our *htrA* knockout strain confirmed a mutation in Pmt, an enzyme responsible for modifying and stabilizing Ami3. Interestingly, this mutation occurred at a site distinct from the traditionally identified conserved site, offering new insights into this crucial mannosyltransferase.

Our data support a model wherein HtrA degrades Ami3, preventing its accumulation to toxic levels. Deletion of *htrA* leads to a dramatic overexpression of Ami3, which in turn inhibits both growth and biofilm formation. A partial growth rescue was achieved by knockdown of *ami3* in the *htrA* knockout strain. The observation that this rescue was incomplete suggests that HtrA possesses biological functions that extend beyond its known interaction with Ami3, such as affecting cell division or elongation. The partial phenotypic rescue by *htrA* complementation is likely attributable to its non-native expression levels from the vector. This is consistent with previous findings that HtrA is essential for degrading Ami3 [32]. The observation that heterologous overexpression of *ami*3_Mtu_ in *M. smegmatis* abolished biofilm formation without affecting growth indicates a specific and conserved role for Ami3 in regulating biofilm development, separable from its general toxicity at high levels. During growth curve analysis, the *htrA* knockout strain exhibited a growth defect that was restored to the wild-type level in the later phase. However, this restored growth did not rescue its biofilm-deficient phenotype. Consequently, we extended the biofilm observation period to 72 h and confirmed that the absence of biofilm formation in the *htrA* knockout strain remained unchanged. This uncoupling of growth and biofilm phenotypes is a significant finding, suggesting that Ami3’s function in biofilm disruption can be targeted independently. The key observation is that the biofilm defect in the *htrA* complementary strain can be partially rescued. This result provides compelling functional evidence that the phenotype is directly attributable to the loss of *htrA*, rather than to potential secondary mutations in other genes. HtrA proteases are recognized as crucial factors for bacterial pathogens to survive under stress and overcome host defenses [13,41,42]. They can enhance pathogenicity by disrupting epithelial barriers and facilitating the function of other virulence factors [43,44]. The role of HtrA in biofilms, however, appears to be context-dependent, with studies showing it can either promote or inhibit biofilm formation in different species [32]. Our work adds a new layer to this understanding by delineating a specific HtrA-Ami3 pathway that controls a key persistence mechanism in mycobacteria. This positions HtrA not just as a general stress response protease but as a dedicated regulator of cell wall homeostasis and social behavior in this important genus.

The function of the Ami family proteins as peptidoglycan muramidases is well-established [37]. These enzymes hydrolyze glycosidic bonds within peptidoglycan, specifically cleaving the β-1,4-glycosidic linkage between N-acetylmuramic acid and N-acetylglucosamine in the polysaccharide backbone. Given the known function of Ami family proteins, we established that Ami3 is required for biofilm formation in mycobacteria, as its absence significantly impaired this process. We also investigated the potential role of Ami2, which was modestly downregulated in the Δ*htrA* strain. However, modulating *ami2* expression had no impact on growth or biofilm, either in a WT or Δ*ami3* background. This suggests that Ami2 does not play a major role in the HtrA-dependent regulatory circuit governing biofilm formation, underscoring the specific role of Ami3 in this process.

Consistent with earlier reports that *htrA* is essential without compensatory mutations [32], we identified novel mutations in Pmt (F53V, N55E) in Δ*htrA* strain. Only after introducing functional mutations in *pmt*, *ami3*, *mprA*, and *mprB* was it possible to knock out *htrA*. Sequencing of knockout strains revealed that only the *pmt* carried a mutation. Differs from traditional loss-of-function mutations that severely disrupt protein activity. These mutations are distinct from the previously characterized D68A mutation [39,42], which is located in the catalytic aspartate residue. Intriguingly, our structural prediction suggests that these new mutation sites (F53, N55) and the known site (D68) are spatially arranged in an approximately symmetrical configuration within the Pmt protein. This raises the possibility that these mutations may disrupt Pmt function in a similar manner, potentially by reducing its ability to mannosylate and stabilize Ami3 as suggested by AlphaFold3 for predictive analysis (Figure S5). By preventing Ami3 stabilization, these *pmt* mutations would counteract the toxicity caused by Ami3 accumulation in the absence of HtrA, thereby explaining their compensatory effect. The existence of multiple, distinct suppressor mutations in *pmt* highlights the critical nature of the functional relationship between HtrA, Pmt, and Ami3 in maintaining cell wall integrity.

In conclusion, this study reveals a novel regulatory axis in which HtrA modulates mycobacterial biofilm formation by controlling the stability of Ami3. We further identify new compensatory mutations in *pmt* that alleviate the essentiality of HtrA for normal growth, providing fresh insights into the genetic network governing cell wall homeostasis. Given that biofilms contribute to antibiotic tolerance and persistence in *M. tuberculosis*, the HtrA-Ami3 interface represents a potential target for novel therapeutic strategies aimed at disrupting biofilm-associated infections.

## 4. Materials and Methods

### 4.1. Strains, Plasmids, and Growth Conditions

Bacterial strains, plasmids, and primers used in this study are listed in Supplementary Tables S3–S5. All primers were synthesized by Tsingke Biological Technology Co., Ltd., Beijing, China. *Escherichia coli* strains were grown in LB liquid medium (10 g/L tryptone (OXOID, Basingstoke, UK), 10 g/L sodium chloride (Sangon Biotech, Shanghai, China), 5 g/L yeast powder (OXOID, Basingstoke, UK) supplemented with an additional 0.015 g/mL agar powder (Solarbio, Beijing, China) for the solid medium. *M. smegmatis* strains were grown in 7H9 liquid medium (4.7 g/L Middlebrook 7H9 (BD Difco, Becton, Dickinson and Company, Franklin Lakes, NJ, USA), 2.5 mL/L 20% Tween80 (Sangon Biotech, Shanghai, China), 4 mL/L 50% glycerol (Sangon Biotech, Shanghai, China) or 7H10 solid medium (1.9 g/100 mL Middlebrook 7H10 (BD Difco, Becton, Dickinson and Company, Franklin Lakes, NJ, USA), 1 mL/100 mL 50% glycerol).

### 4.2. Construction of the Deletion Mutants and Complementation Mycobacteria

The *htrA* and *ami3* gene were knocked out in *M. smegmatis* using a gene replacement strategy Via homologous recombination. Upstream and downstream flanking regions of *htrA* and *ami3* were amplified using the primer pairs listed in Table S3. The resulting PCR fragments were cloned into a pMind-derived suicide plasmid that carried a hygromycin resistance gene and a *lacZ* selection market. The knockout of *htrA* was verified by PCR (Figure S6). Subsequently, these recombinant plasmids were inserted into mycobacteria by transformation.

### 4.3. Colony Morphology Assay

*M. smegmatis* strains were grown to a mid-log phase (OD_600_ 1.0–1.2) in 7H9 medium. For spot colony morphology, 2 μL of cultures was spotted onto 7H10 agar plates and incubated at 37 °C for 3–5 days to observe colony morphology. For single colony morphology, cultures were adjusted to a concentration of 10^6^ and 100 μL were plated on 7H10 agar incubated at 37 °C for 3–5 days before imaging.

### 4.4. Biofilm Phenotyping Experiments

For air-liquid surface biofilm growth assay, strains were grown using 7H9 medium to OD_600_ of 1.0, and after collection, M63 medium (100 mM KH_2_SO_4_, 75 mM KOH, 15 mM (NH_4_)_2_SO_4_ (Solarbio, Beijing, China), 2 mM MgSO_4_ (Sinopharm, Beijing, China), 3.9 μM FeSO_4_ (Solarbio, Beijing, China), 2% glucose (Solarbio, Beijing, China) (m:V), 0.5% casein hydrolysate (Solarbio, Beijing, China) (m:V), 0.7 Mm CaCl_2_ (Solarbio, Beijing, China)) adjusted to OD_600_ of 0.3 and added to 12 well cell well plates (3 mL/well) for 30 °C culture observation. For crystal violet staining quantitative assay, strains were grown to OD_600_ of 1.0 using 7H9 medium, adjusted to OD_600_ of 0.1 using M63 medium after harvest, and added to 96 well cell well plates (100 μL/well) at 37 °C for 80 rpm for 36 h, after which the supernatants were left to dry and 120 μL/well 0.1% (m:V) crystal violet was stained for 30 min at 200 μL/well single distilled water and washed twice, air dried, and added to 200 μL/well ethanol/acetone mixture (80% ethanol (Sangon Biotech, Shanghai, China): 20% acetone (Sangon Biotech, Shanghai, China), (*v*:*v*) was dissolved for 5 min at room temperature, and the absorbance value was detected at 570 nm.

### 4.5. Growth Curve Measurement

Strains were pre-cultured in 7H9 medium with 30 μg/mL Kanamycin to an OD_600_ of ~1.0. The cultures were then harvested, washed, and diluted to a starting OD_600_ of ~0.15 in fresh 7H9 medium. A 200 µL aliquot of each dilution was loaded into a 96-well plate. Growth was monitored by measuring the OD_600_ every 4 h over a 24–48 h period using a plate reader. The plates were incubated at 37 °C or 42 °C with continuous shaking at 150 rpm.

### 4.6. Proteomic Assays

The wild-type and knockout strains were cultured with 7H9 medium to OD_600_ of 1.0, harvested and adjusted to OD_600_ of 0.1, incubated at 37 °C, 150 rpm, until the wild-type strain was grown to OD_600_ of 1.0, collected equally in weight, cleaned with 1× PBS for 2–3 times, and the appropriate amounts were equally divided after collection.

Samples were retrieved from −80 °C storage. An appropriate amount of tissue sample was weighed into a mortar pre-cooled with liquid nitrogen and thoroughly ground to a powder under liquid nitrogen. For each sample, a 4-fold volume of lysis buffer (8 M urea, 1% protease inhibitor) was added, followed by sonication for cell disruption. The homogenate was centrifuged at 12,000× *g* for 10 min at 4 °C to remove cellular debris. The supernatant was transferred to a new centrifuge tube, and protein concentration was determined using a BCA assay kit (Thermo Fisher Scientific, Rockford, IL, USA).

Equal amounts of protein from each sample were taken for digestion. The volume of each sample was adjusted to be consistent using lysis buffer. Trichloroacetic acid (TCA) was slowly added to a final concentration of 20%, followed by vortex mixing. Proteins were precipitated at 4 °C for 2 h. The precipitate was pelleted by centrifugation at 4500× *g* for 5 min, and the supernatant was discarded. The pellet was washed 2–3 times with pre-cooled acetone. After air-drying, the pellet was dissolved in 200 mM triethylammonium bicarbonate (TEAB) buffer and dispersed by sonication. Trypsin was added at a 1:50 (*w*/*w*, enzyme: protein) ratio for overnight digestion. Dithiothreitol (DTT) was then added to a final concentration of 5 mM, and samples were incubated at 56 °C for 30 min for reduction. Subsequently, iodoacetamide (IAA) was added to a final concentration of 11 mM, and samples were incubated at room temperature in the dark for 15 min.

Peptides were dissolved in mobile phase A and separated using an EASY-nLC 1200 (Thermo Fisher Scientific, San Jose, CA, USA) ultra-high performance liquid chromatography (UHPLC) system. Mobile phase A consisted of 0.1% formic acid in water with 2% acetonitrile. Mobile phase B consisted of 0.1% formic acid in acetonitrile with 90% acetonitrile. The LC gradient was set as follows: 0–68 min, 6% to 23% B; 68–82 min, 23% to 32% B; 82–86 min, 32% to 80% B; 86–90 min, 80% B. The flow rate was maintained at 500 nL/min. Peptides separated by UHPLC were ionized using a NanoSpray Ionization (NSI) source and analyzed by an Orbitrap Exploris™ (Thermo Fisher Scientific, San Jose, CA, USA) 480 mass spectrometer. The ion source voltage was set to 2.3 kV, and FAIMS (High-Field Asymmetric Waveform Ion Mobility Spectrometry) compensation voltages (CV) were set to −45 V and −65 V. Both peptide precursor ions and their fragment ions were detected and analyzed using the high-resolution Orbitrap analyzer (Thermo Fisher Scientific, Bremen, Germany). The primary mass spectrometry (MS1) scan range was set to 400–1200 *m*/*z* with a resolution of 60,000. The secondary mass spectrometry (MS2) scan range started fixed at 110 *m*/*z* with a resolution of 15,000. TurboTMT was set to Off. Data acquisition utilized a data-dependent acquisition (DDA) mode: following each full MS1 scan, the top 25 most intense precursor ions were sequentially selected for higher-energy collisional dissociation (HCD) fragmentation with 27% normalized collision energy (NCE), followed by MS2 analysis. To improve effective MS utilization, the Automatic Gain Control (AGC) target was set to 100%, the signal threshold for triggering MS2 scans was set to 5E4 ions/s, the maximum injection time was set to Auto, and a dynamic exclusion duration of 20 s was applied to prevent repeated sequencing of the same precursors.

### 4.7. AlphaFold3 Analyses

The three-dimensional structure of Pmt was predicted using AlphaFold3 Via the publicly available server. The input was the full-length amino acid sequence of Pmt. The model with the highest average pLDDT score was selected. Confidence metrics (pLDDT and PAE) were analyzed to identify well-defined and potentially disordered regions.

### 4.8. CRISPRi Technology

CRISPRi technology is based on CRISPR-Cas9 technology by catalytically inactivating Cas9 to lack endonuclease activity (dCas9) [45]. Target genes can be effectively silenced when dCas9 is co-expressed with sgRNAs designed with a 20 bp complementary region to any gene of interest. The sgRNAs targeting the gene of interest were cloned into the dCas9 vector pLJR962 (Figure S7).

A genome-wide sgRNA library was constructed by designing different sgRNAs targeting the entire *M. smegmatis* genome. The corresponding primer pairs (F and R) were annealed and amplified by PCR, and the resulting fragments were ligated into the pLJR962 plasmid (Figure S7). The library was then transformed into *M. smegmatis*, and transformants were selected on 7H10 medium. Following selection, specific colonies were subjected to repeated screening and sequencing to identify the genomic targets of the sgRNAs. For the targeted knockdown of *ami3*, an *ami3*-targeting sgRNA was cloned into pLJR962, which was then transformed into the *htrA* knockout strain of *M. smegmatis* to generate a mutant strain. Gene repression in phenotypic assays was induced by the addition of 150 ng/mL anhydrotetracycline (ATc) to phenotypic assays.

### 4.9. Statistics

Statistical analysis was performed using GraphPad Prism 7 (GraphPad Software Inc., San Diego, CA, USA). All the data shown are mean ± standard deviation (SD) from at least three biological replicates. Significance testing was by Student’s *t*-test. *p* < 0.05 was considered statistically significant.

## Data Availability

The original contributions presented in this study are included in the article/Supplementary Material. Further inquiries can be directed to the corresponding author.

## Supplementary Materials

The following supporting information can be downloaded at: https://www.mdpi.com/article/10.3390/microorganisms13122688/s1, Table S1: Up-regulated proteins in ΔhtrA compared to WT; Table S2: Down-regulated proteins in ΔhtrA compared to WT; Table S3: Bacterial strains used in this study; Table S4: Plasmids used in this study; Table S5: Primers and ssDNA used in this study; Figure S1: Crystal violet staining of the wild-type, knockout, and complementation strains; Figure S2: The biofilms of wild-type strain, ΔhtrA mutant, and complementation strain (ΔhtrA-comp) were observed following a 72-h incubation period; Figure S3: Effect of ami3 knockdown on the growth of htrA knockout strains; Figure S4: Quantification of biofilm of the wild-type, ΔhtrA and ami3 knockdown strains; Figure S5: The structure of PmtMsm was predicted using AlphaFold3; Figure S6: PCR analysis verifying the knockout of htrA; Figure S7: Schematic representation of the knock down vector pLJR962.
