# Supplementary material for: HtrA Contributes to Biofilm Formation in Mycobacterium smegmatis by Downregulating the Cell Wall Amidase Ami3
Source: Microorganisms. 2025 Nov 25;13(12):2688. doi: 10.3390/microorganisms13122688 (PMC12735335; doi:10.3390/microorganisms13122688)
Supplement: Supplementary file 1 [file microorganisms-13-02688-s001.zip › microorganisms-3966235-supplementary.pdf]

Supplementary Materials

HtrA Contributes to Biofilm Formation in *Mycobacterium smegmatis* by Downregulating the Cell Wall Amidase Ami3

Jiachen Zheng †, Yueqi Li †, Yizhang Wei, Kang Li, Jie Lu, Xiaolin Liu * and Weihui Li *

Guangxi Research Center for Microbial and Enzyme Engineering Technology, State Key Laboratory for Conservation and Utilization of Subtropical Agro-Bioresources, College of Life Science and Technology, Guangxi University, Nanning 530004, China

* Correspondence: xiaolinliu@gxu.edu.cn (X.L.); lwhlbx@163.com (W.L.); Tel.: +86-771-2852965

† These authors contributed equally to this work.

A list of the supplementary materials:

Table S1. Up-regulated proteins in Δ*htrA* compared to WT.

Table S2. Down-regulated proteins in Δ*htrA* compared to WT.

Table S3. Bacterial strains used in this study.

Table S4. Plasmids used in this study.

Table S5. Primers and ssDNA used in this study.

Figure S1. Crystal violet staining of the wild-type, knockout, and complementation strains.

Figure S2. The biofilms of wild-type strain, Δ*htrA* mutant, and complementation strain (Δ*htrA-comp*) were observed following a 72-hour incubation period.

Figure S3. Effect of *ami3* knockdown on the growth of *htrA* knockout strains.

Figure S4. Quantification of biofilm of the wild-type, Δ*htrA* and *ami3* knockdown strains

Figure S5. The structure of Pmt_{Msm} was predicted using AlphaFold3

Figure S6. PCR analysis verifying the knockout of *htrA*

Figure S7. Schematic representation of the knock down vector pLJR962

Table S1. Up-regulated proteins in Δ*htrA* compared to WT.

Up-Regulated	Ratio	Protein Description
MSMEG_5526	64.177	Peptidoglycan-binding LysM
katG1	22.43	Catalase-peroxidase 1
MSMEG_6919	16.708	Proline-rich 28 kDa antigen
MSMEG_2432	10.787	D-alanyl-D-alanine carboxypeptidase
MSMEG_1176	10.707	PE-PPE domain-containing protein
mimR	8.933	Propane 2-monooxygenase operon transcriptional activator
MSMEG_2808	8.026	Short-chain dehydrogenase/reductase SDR
ripA	7.718	Peptidoglycan endopeptidase
MSMEG_3146	7.323	Invasin 1
MSMEG_6263	5.993	Glutamate synthase family protein
MSMEG_2343	5.787	Methylesterase
MSMEG_2584	5.704	Penicillin-binding protein, putative
MSMEG_0242	5.683	MHB domain-containing protein
MSMEG_6383	5.23	Transcription regulator FurA
MSMEG_2107	5.096	DUF4185 domain-containing protein
MSMEG_1629	4.924	Peptidase S15(predicted)
MSMEG_1012	4.694	Alkaline phosphatase
MSMEG_1322	4.487	ErfK/YbiS/YcfS/YnhG family protein
MSMEG_2973	4.286	Peptidyl-prolyl cis-trans isomerase
cydB	3.943	Cytochrome D ubiquinol oxidase, subunit II
MSMEG_2599	3.91	GntR-family protein transcriptional regulator
MSMEG_5300	3.842	Short-chain type dehydrogenase/reductase
MSMEG_1661	3.754	D-alanyl-D-alanine carboxypeptidase
MSMEG_4596	3.75	PknH_C domain-containing protein
MSMEG_1770	3.476	CsbD domain-containing protein
MSMEG_2924	3.419	Permease binding-protein component
MSMEG_1418	3.302	RNA polymerase ECF-type sigma factor
MSMEG_6737	3.242	Putative membrane protein
MSMEG_6497	3.22	Uncharacterized protein
MSMEG_3536	3.078	Sugar transport protein
MSMEG_6281	3.039	N-acetylmuramoyl-L-alanine amidase

Table S2. Down-regulated proteins in Δ*htrA* compared to WT.

Down-Regulated	Ratio	Protein Description
tatB	0.014	Sec-independent protein translocase protein TatB
MSMEG_1446	0.032	NTP pyrophosphohydrolase
MSMEG_5070	0.044	Trypsin
MSMEG_1711	0.053	ATP binding protein of ABC transporter
MSMEG_2005	0.067	Sugar phosphate isomerase/epimerase
MSMEG_1706	0.077	Xylose transport system permease protein XylH
MSMEG_3008	0.079	Putative sigma 54 type regulator
MSMEG_1017	0.08	Glutaredoxin-like protein NrdH
MSMEG_2478	0.084	Fumarylacetoacetate hydrolase family protein
MSMEG_4692	0.105	Protein of uncharacterized function
MSMEG_6294	0.113	Caib/baif family protein
araA	0.15	L-arabinose isomerase
MSMEG_4693	0.153	Uncharacterized protein
MSMEG_1712	0.164	ABC transporter periplasmic-binding protein YtfQ
MSMEG_5704	0.166	Uncharacterized protein
MSMEG_1885	0.166	2Fe-2S iron-sulfur cluster binding domain protein

MSMEG_3787	0.193	D-aminoacylase
MSMEG_0530	0.198	Short chain dehydrogenase
MSMEG_2162	0.201	MmcJ protein
MSMEG_1254	0.202	DEAD/DEAH box helicase
MSMEG_2100	0.205	Peptidase family protein M20/M25/M40
glnT	0.207	Glutamine synthetase
MSMEG_1155	0.219	Carnitinyl-CoA dehydratase
ectB	0.22	Diaminobutyrate--2-oxoglutarate transaminase
MSMEG_0438	0.221	Periplasmic binding protein
MSMEG_1714	0.221	L-ribulose-5-phosphate 4-epimerase UlaF
MSMEG_1704	0.223	ABC transporter
MSMEG_4986	0.227	Glycosyl transferase, family protein 39
MSMEG_0022	0.232	L-lysine N6-monooxygenase MbtG
MSMEG_3994	0.25	Short chain dehydrogenase
MSMEG_4539	0.263	Alkanesulfonate monooxygenase
MSMEG_2985	0.274	Fumarate hydratase class I
MSMEG_5651	0.275	Transcriptional regulator, LuxR family protein
MSMEG_1026	0.277	Putative acetyltransferase
MSMEG_6424	0.284	Probable conserved transmembrane protein
MSMEG_1154	0.285	Formyl-coenzyme A transferase
MSMEG_2623	0.286	Tat (Twin-arginine translocation) pathway signal sequence domain protein
MSMEG_1764	0.292	Lysine 6-aminotransferase
MSMEG_5102	0.294	ABC transporter ATP-binding protein

Table S3. Bacterial strains used in this study.

Strain	Description	Source
Escherichia coli		
DH5α	Gene cloning	Laboratory preservation
BL21(DE3)	Protein expression	Laboratory preservation
Mycobacterium smegmatis mc²155		
Msm/WT	WT strain	Laboratory preservation
Msm/pMV261	WT strain with pMV261empty vector	This study
Msm *htrA*::*hyg*/pMV261	*htrA* deletion strain with pMV261 empty vector	This study
Msm *htrA*::*hyg*/pMV261-*htrA*	*htrA* deletion strain with *htrA* complement vector	This study
Msm/pJAM2	WT strain with pJAM2 empty vector	David Albesa-Jové. 2016
Msm/pJAM2-*ami3*	WT strain with *ami3* overexpression vector	This study
Msm *ami3*::*hyg*/pMV261	*ami3* deletion strain with pMV261 empty vector	This study
Msm *ami3*::*hyg*/pMV261- *ami*3	*ami3* deletion strain with *ami3* complement vector	This study
Msm/pJAM2-*ami3* $_{Mtu}$	WT strain with *ami3*$_{Mtu}$ overexpression vector	This study
Msm *ami3*::*hyg*/ CRISPRi-*ami*2	*ami3* deletion strain with *ami*2 knockdown vector	This study
CRISPRi-*ami2*	WT strain with *ami2* knockdown vector	This study

Table S4. Plasmids used in this study.

Plasmid	Purpose	Source
pLJR962	CRISPRi library screening, controls	Laboratory preservation
pMV261	Gene complementation, control	Laboratory preservation
pMV261+	Gene complementation	Laboratory preservation
pMind-*lacZ*-*htrA*	Gene deletion	This study
pMV261-*htrA*	Gene complementation	This study
pJAM2	Gene overexpression, controls	David Albesa-Jové. 2016
pJAM2-*ami*3	Gene overexpression	This study
pMind-*lacZ*-*ami*3	Gene deletion	This study
pJAM2-*ami*3$_{Mtu}$	Gene overexpression	This study
pLJR962-*ami*2	CRISPRi	This study

Table S5. Primers and ssDNA used in this study.

Primer	Sequence(5′→3′)	Purpose
Test1f	ACCGATACCAGGATCTTGCCATCC	CRISPRi strain amplification
Test1r	GTGGCGATAAGTCGTGTCTTACCG	CRISPRi strain amplification
Test2f	TGATGCGCTGGCAGTGTTCCTGCG	CRISPRi strain amplification
Test2r	GTTCGTGCACACAGCCCAGCTTGG	CRISPRi strain amplification
Test3f	CGCTGACTTGACGGGACGGCGGCT	CRISPRi strain amplification
Test3r	AAGCGCCACGCTTCCCGAGGGGAG	CRISPRi strain amplification
*htrA*upf	ATATTTAATTAACTCGCCTACCGCTTGTCGGGC	Gene deletion
*htrA*upr	ATATACTAGTCGGTGTTTCACACTCTCTATCGG	Gene deletion
*htrA*downf	ATATAAGCTTCGATGTTCGCCAACATCGGGTG	Gene deletion
*htrA*downr	ATATGCTAGCACGTGAAGGTCATCTCGATCG	Gene deletion
*htrA*f	ATATGAATTCATGTGACCAACCAGGAACAGTCC	Gene cloning
*htrA*r	ATATTCTAGATTACTGGGCTTTTTGGTCGTCGC	Gene cloning
*hyg*f	AGCCAGCGCATATGGTGACACAAGAATCCCTG	Gene deletion validation
*hyg*r	ACACTTAATTAATTAGGCGCCGGGGGCGGT	Gene deletion validation
*ami*3f1	CCGTGTACGACGCGGGGAACCGGGCCGGCATCGTGC	Gene point mutations
*ami*3r1	GCACGATGCCGGCCCGGTTCCCCGCGTCGTACACGG	Gene point mutations
*ami*3f	ATATGGATCCGTGCAGTCACGTCGTCCCGC	Gene cloning
*ami*3r	GCGTTCTAGATCAGGCGATCGGCGTGAAGC	Gene cloning
*ami*3upf	ATATTTAATTAACGCACCCCAACACCGCGAGT	Gene deletion
*ami*3upr	ATATACTAGTCACGGACTGATGTTACGTA TGCG	Gene deletion
*ami*3downf	GCGCAAGCTTGCCGAAAAGCCCGAAAAGCC	Gene deletion
*ami*3downr	ATATGCTAGCAGAGGCCAAACGCCGCGACG	Gene deletion
*ami*3f(com)	ATATGAATTCAGTGCAGTCACGTCGTCCCGCG	Gene cloning
*ami*3r(com)	ATATTCTAGATCAGGCGATCGGCGTGAAGCGC	Gene cloning
*ami*3$_{Mtu}$f	ATATGGATCCATGGCAGCGACCGTCGTCATC	Gene cloning
*ami*3$_{Mtu}$r	ACGCTCTAGATCAGGTGATCGGATGCGTTGG	Gene cloning
*ami*2sgf	GGGACGGGGCACCGAACTGGTGCG	CRISPRi
*ami*2sgr	AAACCGCACCAGTTCGGTGCCCCG	CRISPRi

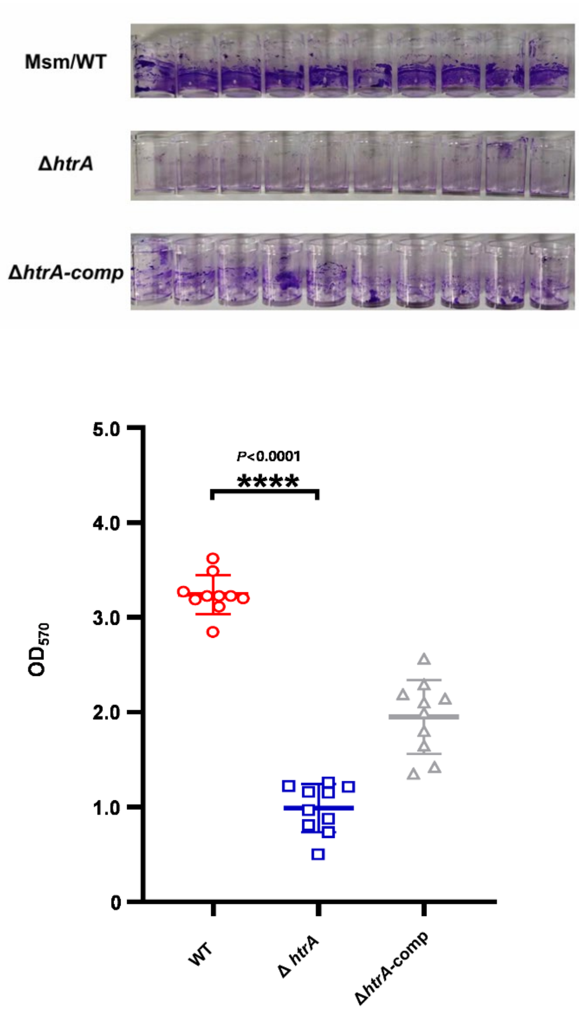

Figure S1. Quantitation of biofilm biomass by crystal violets staining of the wild-type, Δ*htrA*, and complementation strains(Δ*htrA*-comp). (Top panel) Crystal violet staining of biofilms formed by the wild-type (WT), Δ*htrA*, and complemented (Δ*htrA-comp)* strains after 36 hours of growth in 96-well plates. (Bottom panel) Quantitative analysis of biofilm biomass measured by crystal violet elution at OD_{570}. Data represent mean ± SD from ten replicates (n=10). Statistical significance was determined by Student's *t*-test .

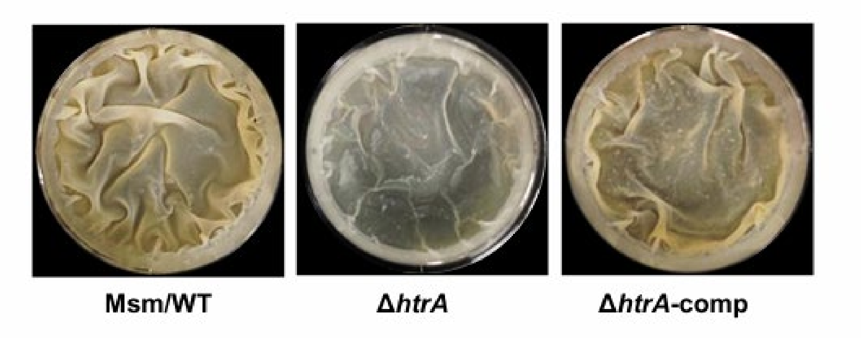

Figure S2. The biofilms of wild-type strain, Δ*htrA* mutant, and complementation strain (Δ*htrA-comp*) strains **were observed following a 72-hour incubation period.** For air-liquid surface biofilm growth assay, wild-type, Δ*htrA*, and complementation strains (Δ*htrA-comp*) strains were grown using 7H9 medium to OD_{600} of 1.0, and after collection, M63 medium adjusted to OD_{600} of 0.3 and added to 12 well cell well plates (3 mL/well) for 30℃ culture observation.

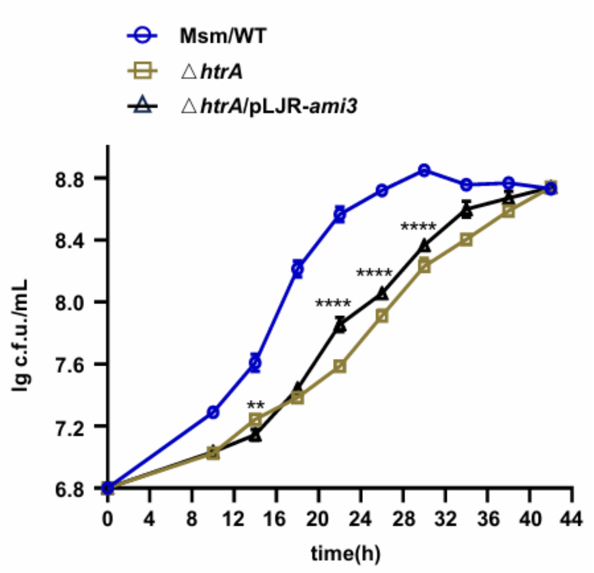

Figure S3. Effect of *ami3* knockdown on the growth of *htrA* knockout strains. Strains were pre-cultured in 7H9 medium with 30 μg/ml Kanamycin to an OD_{600} of ~1.0. The cultures were then harvested, washed, and diluted to a starting OD_{600} of ~0.15 in fresh 7H9 medium. A 200 μL aliquot of each dilution was loaded into a 96-well plate. Growth was monitored by measuring the OD_{600} every 4 hours over a 24-48 hour period using a plate reader. The plates were incubated at 37℃ or 42℃ with continuous shaking at 150 rpm.

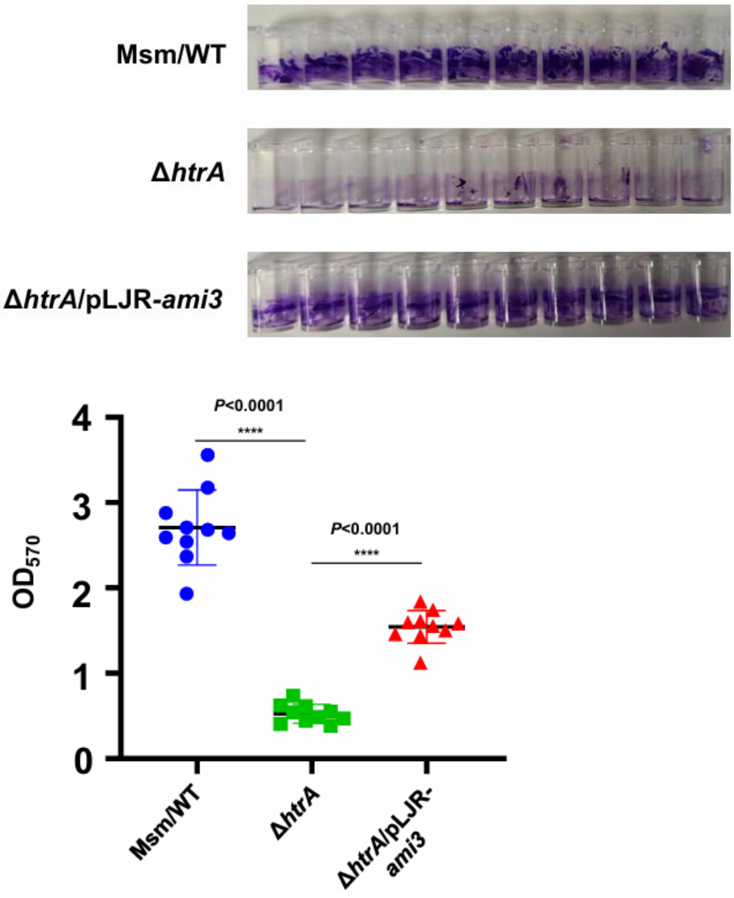

Figure S4. Biofilm quantification confirms partial rescue in the Δ*htrA* strain by *ami3* knockdown. Strains were grown to OD_{600} of 1.0 using 7H9 medium, adjusted to OD_{600} of 0.1 using M63 medium after harvest, and added to 96 well cell well plates (100 μL/well) at 37℃ for 80 rpm for 36 h.

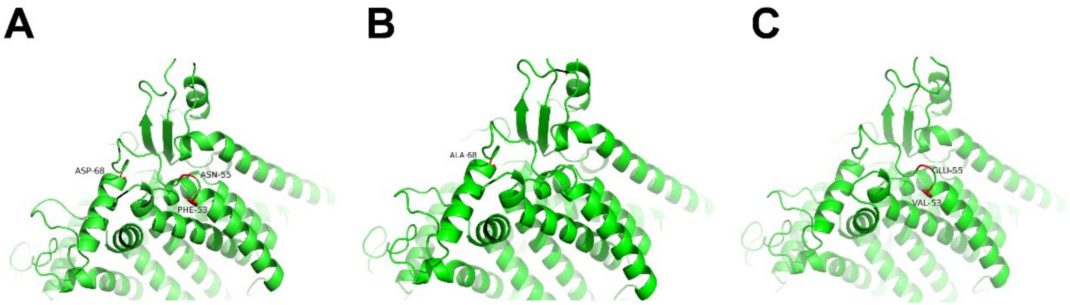

Figure S5. The structure of Pmt$_{Msm}$ was predicted using AlphaFold3 (via the AlphaFold Server). Input sequences in FASTA format were submitted without providing any template structures. The model with the highest predicted confidence ipTM=0.93 (The structure was predicted by AlphaFold3. The interface predicted TM-score (ipTM), indicating a highly reliable prediction.) was selected for further analysis. All structural visualization, analysis, and figure generation were performed using PyMOL.

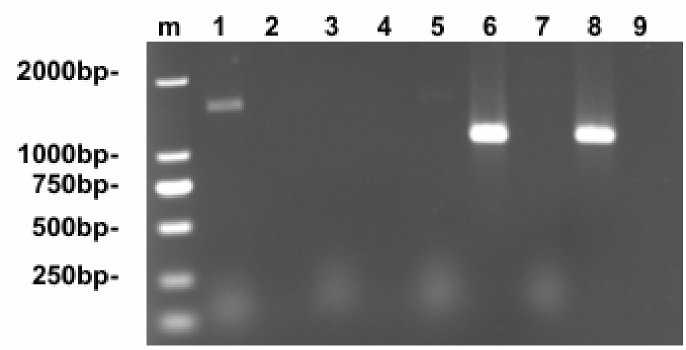

Figure S6. PCR analysis verifying the knockout of *htrA*. The gel image shows the amplification products from wild-type (WT) and mutant DNA. line 1: PCR amplification of the *htrA* gene from a wild-type template; line 2-4: PCR amplification of the *htrA* gene from a *htrA* knockout strainse template; line 5: PCR amplification of the *htrA* gene from water; line 6-8: PCR amplification of the *hyg* gene from a *htrA* knockout strain template; line 9: PCR amplification of the *hyg* gene from water.

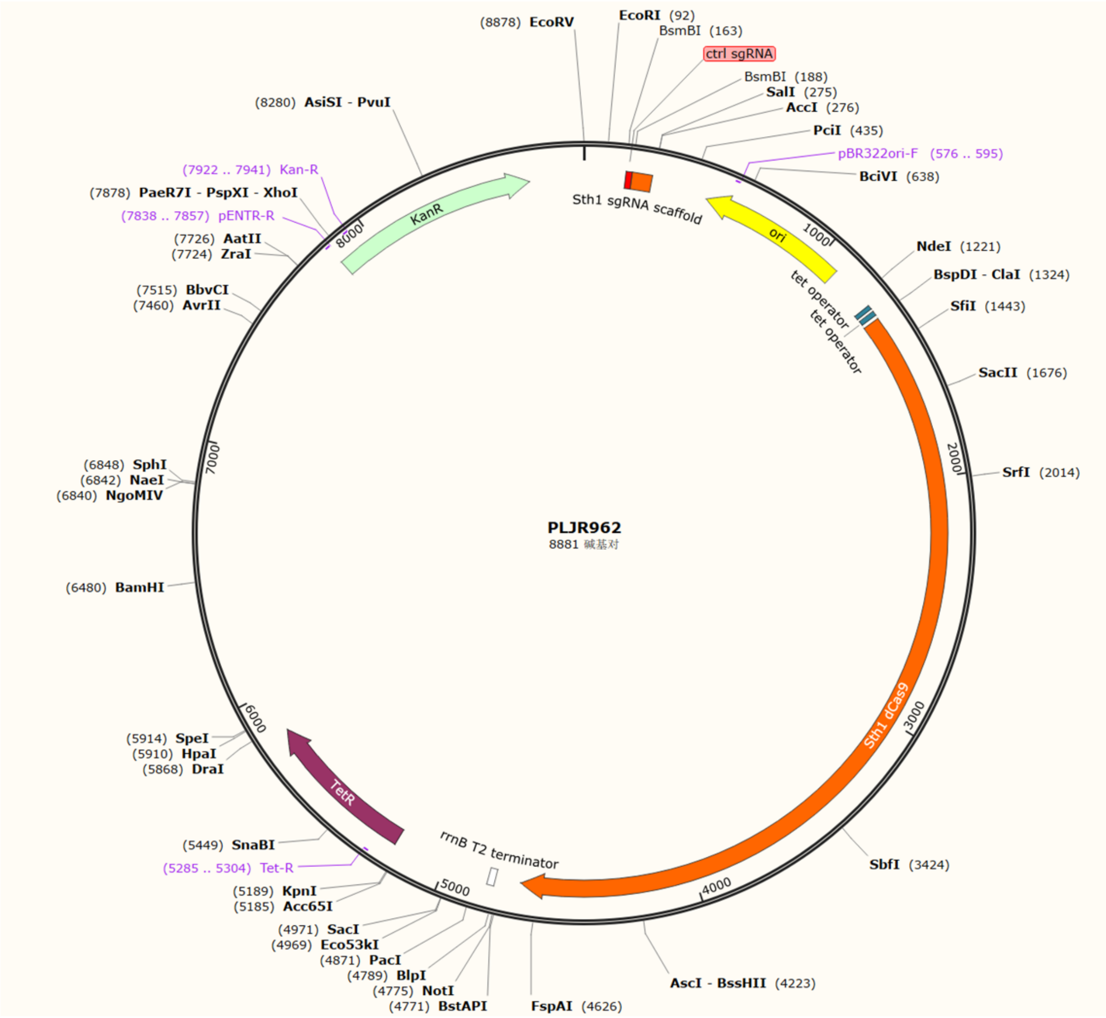

Figure S7. Schematic representation of the knock down vector pLJR962.
